# Mesangial Cells in Diabetic Kidney Disease: From Mechanisms to Therapeutic Implications

**DOI:** 10.7150/ijbs.114907

**Published:** 2025-07-24

**Authors:** Li Feng, Yu-ying Feng, Qian Ren, Ping Fu, Liang Ma

**Affiliations:** Department of Nephrology, Institute of Kidney Diseases, West China Hospital of Sichuan University, and National Key Laboratory of Kidney Diseases, Chengdu 610041, China.

**Keywords:** diabetic kidney disease, mesangial cells, pathophysiology, drug target.

## Abstract

Diabetic kidney disease (DKD) is a serious diabetic complication, the morbidity and mortality of which has rapidly increased worldwide. As known, the pathogenesis of DKD is complex and includes mesangial cell (MC) proliferation and hypertrophy, mesangial expansion, glomerular basement membrane thickening, podocyte detachment, and tubulointerstitial damage. Thus, the role of MCs cannot be underestimated, as their exclusive positioning and diverse physiological functions are crucial for preserving the glomerular filtration membrane's composition and functionality. Considerable animal studies and clinical trials have elaborated that MCs are pivotal to the occurrence and progression of DKD. In this review, we summarize and outline the mechanisms of MC injury and the interactions of MCs with other cells and discuss the progress of MC-targeted therapeutics to provide a comprehensive perspective on the prevention and treatment of DKD.

## Introduction

Diabetes is a global metabolic disease. More than 10.5% of adults worldwide have diabetes 1-6. Epidemiological studies have demonstrated that approximately 40% of diabetic patients ultimately progress into diabetic kidney disease (DKD), with significant variations among different geographical regions and ethnic groups 7. Notably, African-descent and Asian populations exhibit particularly elevated disease susceptibility, whereas low-income countries bear a disproportionately heavier disease burden 8. Geographically, DKD has consistently remained the leading etiology of end-stage renal disease (ESRD) in developed Western countries. Concurrently, epidemiological data from China have revealed a steady upward trajectory in DKD incidence, establishing it as one of the principal causes of kidney failure (KF) 9. As the incidence of diabetes has increased worldwide, DKD has become highly prevalent and is a major cause of heart disease, early death, and worldwide health care expenditures 10. However, the etiology of DKD is complex, and various pathophysiological mechanisms might contribute to DKD, including abnormalities in glomerular hemodynamic function, oxidative stress, mitochondrial dysfunction, and the activation of proinflammatory and profibrotic factors 11. Moreover, multiple cellular abnormalities are responsible for the development and progression of DKD.

Glomerular endothelial cell dysfunction, mesangial cell (MC) damage, mesangial matrix accumulation, podocyte shedding, and tubular epithelial injury are involved in the process of renal fibrosis, which eventually lead to the renal impairment in DKD patients 12, 13. Here, the MC injury is a significant pathogenic process during the development of DKD. MCs are widely known to maintain homeostasis under healthy conditions, with dynamic balancing of the synthesis and degradation of mesangial matrix proteins. Under stable conditions, MCs preserve the normal structure of the surrounding capillary network and ensure that the glomerulus can perform its filtering function 14. However, when exposed to certain pathological factors and injury-inducing stimulations, such as hyperglycemia, hypertension, hyperlipidemia, reactive oxygen species (ROS), inflammatory factors, and so on, MCs are activated and both proliferate and secrete cytokines and matrix proteins, ultimately leading to the loss of glomerular capillary loops and glomerular function. Aberrant proliferation of MCs and excessive matrix expansion have been identified as pivotal pathological features in diseases such as IgA nephropathy and lupus nephritis 15, 16. These processes contribute to renal fibrosis progression by obstructing Bowman's capsule, disrupting the filtration barrier, and ultimately leading to glomerulosclerosis and loss of renal function.

Additionally, MCs could directly contact with endothelial cells and separate from podocytes via the glomerular basement membrane (GBM). This architectural framework supports the necessary conditions for physiological intercellular communication within the glomerulus. Emerging evidence has indicated that kidney cells and the interaction between kidney cells and immune cells play pivotal roles in triggering the course of DKD 17. This complex interaction is notably visible in endothelial dysfunction, mesangial enlargement, podocyte injury, and progressive podocyte apoptosis, which can exacerbate glomerulosclerosis and functional degradation 18. Accumulating, studies have highlighted that MCs not only maintain the stability of the capillary network, but also receive and release a range of growth factors and cytokines to play a signaling role 19-21. Therefore, further exploration of the interactions between MCs and other cells is highly important.

In this review, we examine the molecular mechanisms of MC damage and create a web of cellular crosstalk focused on MCs to investigate their roles in DKD. Importantly, we also discuss the MC-targeted treatment strategies for DKD.

## Pathophysiology of MC damage in DKD

The central axis of the glomerulus is made up of MCs and their mesangial stroma, which are spread throughout the capillary network and, along with endothelial cells and podocytes, maintain the normal glomerular structure. In their resting state, MCs exhibit a stellate morphology; however, upon activation, they transform into elongated spindle-shaped cells closely resembling fibroblasts. 22. MCs have cellular protrusions that extend from the cell's center of the cell into the surrounding capillary lumen, allowing them to perform a range of physiological activities 23. In DKD, MCs undergo a significant phenotypic shift from a quiescent state to a dedifferentiated state with elevated α-SMA expression. MCs undergoing phenotypic transition exhibit robust secretory capacity, releasing substantial quantities of inflammatory mediators and pro-fibrotic factors. These secretory products collectively contribute to renal pathology in DKD through both autonomous mechanisms and paracrine interactions with neighboring cell populations. 24. However, much remains undiscovered in the context of MC damage in DKD. With the progression of biomedical research, substantial scientific evidence has elucidated the molecular mechanisms underlying MCs injury in DKD pathophysiology. The following part will focus on the molecular mechanisms underlying DKD MC failure in DKD from four perspectives.

### Oxidative stress in MCs

Oxidative stress arises from a disruptive imbalance between pro-oxidant and antioxidant forces within biological systems, triggering the relentless buildup of reactive oxygen species (ROS) and reactive nitrogen intermediates. This biochemical cascade ultimately inflicts oxidative injury upon vital biomolecules, compromising DNA integrity and denaturing functional proteins. 25. The pathogenesis of DKD is complex, among which oxidative stress is particularly important 26. Many studies suggest that MC exposure to a hyperglycemic environment increases the levels of ROS and inflammatory mediators, which may be strongly related to DKD 27. The accumulation of ROS can lead to mesangial dysfunction, an imbalance of mesangial matrix synthesis and degradation, and cause glomerulosclerosis and loss of filtration function. It has been revealed that Fyn, a member of the Src kinase, is engaged in the oxidative stress associated with of acute renal damage 28. Knockdown of Fyn reduced high-glucose-induced ROS levels in MCs and DKD kidney tissue, improving renal fibrosis 29. Another study reported that overexpression of the transcription factor FOXO1 reduced the ROS accumulation in MCs under high-glucose stimulation 30. Nuclear factor erythroid 2-related factor (Nrf2) is the main regulator of cells maintaining redox homeostasis and plays an antioxidant role 31. Studies have shown that the expression of Nrf2 and its target gene expression are downregulated in DKD, and that activation of Nrf2 could improve high-glucose triggered oxidative stress and inflammation in MCs 32. Research has demonstrated that the transcription factor YY1 promotes renal fibrogenesis in DKD through activation of the HIF-1α/mROS signaling pathway. Mechanistically, under HG conditions, YY1 increases hypoxia-inducible factor-1α (HIF-1α) expression and activity in MCs, leading to excessive mitochondrial ROS (mROS) production. Furthermore, mROS reciprocally enhances YY1/HIF-1α signaling activation, establishing a positive feedback loop that ultimately drives MC hyperproliferation 33.

### Mitochondrial dysfunction in MCs

The factors that cause the development of DKD are not clear, but numerous studies have shown that mitochondrial dysfunction is related to the progression of DKD 34. Mitochondria are extremely important organelles in eukaryotic cells and exert have a wide range of biological functions. Mitochondria, the main site of glucose, lipid, and amino acid metabolism, supply the energy and nutrients required to adjust to different cellular conditions and warning signs. The kidney is an organ with high metabolism and high energy demand, and various biological processes require mitochondria to supply energy. Therefore, mitochondrial function is a necessary condition for maintaining normal kidney structure and function. MCs contain several mitochondria, and the mitochondrial dysfunction of MCs under high-glucose conditions leads to MC damage and promotes the progression of DKD.

#### Mitophagy

Autophagy is a conserved process in which for removing excess organelles and misfolded proteins are removed from the body 35. Autophagosomes encapsulate proteins or organelles that need to be degraded and transport them to lysosomes, where multiple proteases degrade these materials. The degraded products are recycled and reused to synthesize new proteins and organelles 36. Mitophagy is the process of targeting the transfer of damaged or dysfunctional mitochondria to autophagosomes for degradation through lysosomes 37. In DKD, a large number of functionally impaired mitochondria accumulate in the kidney, suggesting that mitophagy function may be impaired 38. Mitophagy is mainly mediated by the PINK1/Parkin pathway 39, which is also inhibited in DKD 36, 38. A study reported that erythropoietin (EPO) could reverse high-glucose-induced inhibition of the PINK1/Parkin pathway in MCs, restore mitophagy in DKD, and ameliorate renal damage 40.

#### Mitochondrial fusion and fission

The process of uniting two mitochondria's inner and outer membranes to create a single mitochondrion is known as mitochondrial fusion, and mitochondrial fission involves the breakage of the inner and outer membranes of one mitochondrion into two mitochondria 41. Fusion and fission are adaptive changes in mitochondria that are used to meet cellular energy needs 42. Mitochondria repair their function by fusing with mitochondrial DNA or proteins, which then divide to form new functional mitochondria, while the remaining defective proteins are recycled via autophagy 43, 44. In DKD, mitochondrial fusion and fission are increased, and mitophagy is impaired, resulting in many defective mitochondria that cannot be recycled and reused, leading to insufficient mitochondrial ATP 44, 45. Dynamic GTPase dynamite-related protein 1 (Drp1) is a mitochondrial cleavage protein that is located in the cytoplasm, translocates to the outer mitochondrial membrane, and promotes mitochondrial fission in a GTP-dependent manner 34. Studies have shown that Rho-associated coiled-coil kinase 1 (ROCK1) in DKD causes mitochondrial fission by promoting the aggregation of Drp1 in mitochondria. Mechanistically, the serine residue at position 600 of Drp1 is phosphorylated by ROCK1, thereby triggering the translocation of Drp1 46. Multiple studies have shown that inhibiting mitochondrial fission caused by Drp1 could improve renal damage in DKD 47. Phosphodiesterase-4 (PDE4) is a member of the phosphodiesterase family and is involved in the progression of DKD 48. PDE4 overexpression can increase the expression of Drp1 and reduce the phosphorylation of Drp1 at serine 637 by inhibiting protein kinase A (PKA), thereby promoting high-glucose-induced mitochondrial fission in MCs 49. Single amino acid phosphorylation of Drp1 can regulate mitochondrial fission and DKD progression, but it seems that different phosphorylation sites play different roles. For example, in rat aortic smooth muscle cells, angiotensin II induces the phosphorylation of Drp1 serine residue 616 to promote mitochondrial fission 50. Elucidating the signaling mechanisms associated with altered mitochondrial dynamics is essential for understanding the pathogenic mechanisms underlying mitochondrial dysregulation during the early stages of hyperglycemic conditions.

#### Mitochondrial biogenesis

Mitophagy, fusion, and fission are all necessary for improved mitochondrial biogenesis which is the process of maintaining the number of mitochondria 51. Mitochondrial DNA (mtDNA) and nuclear DNA (nDNA) jointly regulate mitochondrial biogenesis. In DKD, the reduction in mitochondrial DNA amount, the impairation of mitochondrial biogenesis, the mitochondrial dysfunction, and insufficiency in energy supply altogether, ultimately lead to cell apoptosis. Multiple studies have shown that nuclear respiratory factor 1 (NRF1), peroxisome proliferator-activated receptor gamma coactivator-1α (PGC-1α), and AMP-activated protein kinase (AMPK) are major regulators of mitochondrial biogenesis 52-54. Downregulation of PGC1-α and NRF1 promotes renal injury and dysfunction in DKD 55, 56. The expression and activity of the transcription factor FOXO1 are downregulated in MCs and DKD kidneys stimulated by high glucose 57, 58. Studies have shown that the decreased FOXO1 activity reduces the transcription of its target gene PGC1-α in MCs, thereby inhibiting mitochondrial biogenesis and promoting the progression of DKD 30. Additionally, recent studies have shown that the glucagon-like peptide 1 receptor (GLP-1R) agonist exendin-4 improves DKD by increasing the expression of PGC1-α 59. The natural polyphenol resveratrol was also found to improve the damage to MCs stimulated by high glucose, acting through the AMPK-SIRT1-PGC1α axis 50.

### Lipid metabolism of MCs

Lipid metabolism is severely dysregulated in the early stages of DKD, which has sparked widespread interest. Accumulating evidence has demonstrated the dysfunction of lipid metabolism during DKD. These alterations include increased fatty acid intake, reduced fatty acid utilization, cholesterol accumulation, aberrant lipoprotein levels, and ectopic lipid deposition 60-63. Mitochondrial dysfunction and dysregulated lipid metabolism form a self-perpetuating vicious cycle in DKD. Excessive FFAs induce mitochondrial reactive ROS overproduction and electron transport chain (ETC) impairment via β-oxidation, whereas dysfunctional mitochondria further suppress lipid catabolism due to insufficient ATP generation, exacerbating intracellular lipid accumulation.

Cluster of differentiation 36 (CD36) and FA-binding protein (FABP) increase the absorption of fatty acids, which are subsequently delivered to mitochondria by carnitine palmitoyltransferase (CPT), a rate-limiting enzyme for FA oxidation 64, 65. The misregulation of fatty acid utilization is well-acknowledged as a primary cause of diabetic kidney injury 66. The lipid metabolism of human MCs exposed to hyperglycemia is altered through the combined activation of inflammatory and proliferative pathways 67. According to recent studies, fatty acid oxidation is reduced in DKD MCs and that genetic deletion of ROCK1 leads to the restoration of fatty acid oxidation and mitochondrial function, indicating the detrimental role of deficient fatty acid oxidation in MC damage in DKD 66.

Additionally, several free fatty acids (FFAs) and phospholipids are elevated in DKD and are commonly acknowledged as risk factors of the disease 68. Palmitate, a common saturated fatty acid, caused considerable lipid accumulation, ROS generation, oxidative stress, and fibrosis in human MCs 69. One of the main potential mechanisms is associated with connected to the FFA transporter protein CD36. By facilitating lipid deposition, CD36 significantly increases transient receptor potential canonical channel 6 (TRPC6)-induced activation of nuclear factor of activated T cell 2 (NFAT2), which is crucial for renal fibrosis. In contrast, lysophosphatidic acid (LPA) causes fibrosis in DKD MCs through increasing the expression of carbohydrate-responsive element-binding protein (ChREBP), which is linked to glycolysis and adipogenic metabolism 70. Mechanistically, the binding of LPA to its receptor causes ROS production in cells, upregulates the E3 ubiquitin ligase Traf4, and promotes the ubiquitination of the HECT-type E3 ubiquitin ligase Smurf2, which inhibits ChREBP ubiquitination and increases its expression 70. However, Yao et al. reported that caffeoylisocitric acid activated Nrf2 and inhibited MAPK signaling, which is widely recognized as a significant mediator of oxidative stress, ultimately leading to attenuation of high-glucose-induced oxidative stress in MCs and extracellular matrix accumulation 67.

Research indicates that renal accumulation of cholesterol is linked to an increased risk of DKD 71, 72. Abnormalities in intracellular cholesterol synthesis, uptake, and export cause total cholesterol accumulation in DKD kidneys. DKD MCs present the downregulation of cholesterol transport proteins such as adenosine triphosphate binding cassette transporter A1 (ABCA1), G1 (ABCG1), and type I scavenger receptor class B (SR-BI) 73. In addition, liver X receptor (LXR) agonists, which play crucial roles in the activation of cholesterol transport proteins, ameliorated renal cholesterol accumulation and inflammatory outbreaks via the delivery of synthetic high-density lipoprotein (sHDL) nanodiscs to MCs 74. Apolipoprotein C3 (ApoC3) is a triglyceride regulator. Huan et al. 75 found that ApoC3 exacerbates renal inflammation through activation of the TLR2/NF-κB pathway, leading to the initial development of DKD, and that this effect is independent of the triglyceride-regulating effects of ApoC3. The NF-κB pathway is well known as an inflammatory signaling pathway, and a recent article reported that the accumulation of the inflammatory factor high-mobility group box 1 (Hmgb1) in MCs exacerbated DKD progression by accelerating the activation of the NF-κB signaling pathway through binding to IκBα 76. Future research should focus on the regulatory mechanisms of lipid metabolism in MCs in order to identify and create more promising DKD therapeutics.

### Epigenetics in MCs

Epigenetic modifications are heritable alterations in cellular phenotype that primarily include DNA methylation, histone modifications, and noncoding RNAs and are unaffected by DNA sequence changes. The amount of information demonstrating that epigenetic pathways exert crucial roles in the pathogenesis of diseases, including cancer, liver fibrosis, and osteoarthritis, has gained substantial attention 77-79. However, there is still remains much to explore regarding epigenetic regulation that is needed in the field of kidney diseases, particularly DKD. Due to considerable research and advancements in epigenetic techniques, it is commonly recognized that abnormal gene expression caused by epigenetic modifications is commonly recognized to be associated with MC damage and the consequent loss of renal function in DKD. In the following paragraphs, we will discuss how epigenetic alterations influence MC inflammation, MC matrix secretion, and renal function in DKD.

#### DNA methylation

DNA methylation, a thoroughly researched epigenetic regulatory mechanism involving the transfer of methyl groups to the carbon atom at position 5 of cytosine, occurs predominantly in CpG-rich regions known as CpG islands, which are found in regulatory regions such as promoters or enhancers of genes 80. Compelling evidence suggests that DNA methylation alters the stability, conformation, and chromatin structure of DNA as well as how DNA interacts with proteins to regulate the expression of specific genes 81. Notably, it is difficult for heavily methylated promoter-area CpG islands to bind to transcription factors, resulting in transcriptional arrest; hence, DNA methylation is commonly regarded as a gene silencing mechanism. DNA methylation is strongly associated with DKD. Previous research has shown that C/EBP homologous protein (CHOP) promotes high-glucose-induced apoptosis in MCs 82. Recent research has demonstrated that in DKD, decreased expression of the E3 ligase TRIM13, also known as RFP2, prevents ubiquitinated degradation of CHOP, which promotes mesangial collagen formation. Mechanistically, reduced TRIM13 expression in DKD is largely due to DNA methylation, since TRIM13 was considerably upregulated in MCs treated with a DNA methyltransferase inhibitor (5-Aza-CdR) 83. It is well known that DNA methylation is a reversible process, and high methylation levels catalyzed by DNA methyltransferases (DNMTs) can undergo demethylation in specific cellular environments or diseased states, which makes DNA methylation an extremely promising therapeutic strategy 84-86.

#### Histone modifications

Large molecules of DNA and histone complexes make up the nucleosome, the basic structural unit of chromatin 87. Enzymes can modify the free amino terminus of histones in a variety of ways, including acetylation, methylation, phosphorylation, and ubiquitination 88. These modifications can alter chromatin function, such as by altering the affinity of histones for DNA double strands and affecting the binding of transcription factors to gene promoters, but do not change the DNA sequence 89. Histone methylation is the addition of methyl groups to histone tails by histone methyltransferases (HMTs), typically at arginine or lysine residues. Histone methylation, like DNA methylation, is a dynamically reversible modification process because histone demethylases remove methyl groups from amino acid residues, making histone methylation an important regulator of gene expression. The degree of histone methylation and the location of modification affect whether gene transcription is inhibited or enabled. For example, histone H3 lysine 9 (H3K9) and H3K27 methylation are related to gene silencing, whereas H3K4 methylation is connected with gene activation, which greatly increases the complexity of gene expression regulation. Enhancer of zeste homolog-2 (EZH2), a histone methyltransferase that is overexpressed in the cortex of diabetic mouse kdineys and was able to methylates H3K27, resulting in the transcriptional inhibition of target genes 90. Das et al. 91 discovered that the high-glucose-induced increase in EZH2 expression promoted MC hypertrophy and matrix expansion, and mechanistic studies revealed that this may be because EZH2 promotes lysine-27 trimethylation of histone H3 (H3K27Me3), which induces downregulation of the expression of deptor, a negative regulator of the mTOR complex, and consequently activates the mTORC1 and mTORC2 activities. In contrast, another study revealed that EZH2 expression was downregulated in MCs exposed to high glucose, whereas overexpression of O-linked N-acetylglucosaminyltransferase (OGT) increased EZH2 glycosylation and maintained EZH2 stability to limit DKD progression 92. The differences in the results could be because of the use of different animal strains and cell lines. To summarize, the role of EZH2 in DKD remains contentious and will require further investigation in the future.

Histone acetyltransferase (HAT) and histone deacetylase (HDAC) regulate histone acetylation, which mostly takes place at the lysine residues of H3 and H4. Acetylation can influence gene transcription by changing the histone charge and interacting with proteins. In general, DNA is negatively charged, but histones are positively charged, causing DNA and histones to bind closely. HAT adds acetyl groups to lysine residues, neutralizing the positive charge of histones, resulting in weaker connections with DNA and promoting transcription 93. Thus, it is widely acknowledged that histone acetylation is related to gene expression, whereas deacetylation is involved in the suppression of gene transcription 94. Histone acetylation is strongly linked to the development of DKD. Vega et al. 95 discovered that lysine acetylation facilitated high-glucose-induced fibronectin (FN) assembly in MCs, as FN assembly increased considerably in MCs exposed to HDAC. However, other forms of histone modifications, such as ubiquitination and phosphorylation, have received less attention in DKD MCs. The prospective significance of histone modifications in DKD MCs is not fully understood.

#### Regulation of noncoding RNAs

Noncoding RNAs, or RNAs that cannot be translated into proteins, such as microRNAs (miRNAs), long noncoding RNAs (lncRNAs), and circular RNAs (circRNAs), have been identified as epigenetic regulators and are involved in controlling a variety of biological processes in the development of DKD, including mesangial proliferation, fibrosis, and matrix secretion. The miRNAs are small, highly conserved RNAs composed of 18-25 nucleotides that regulate gene expression by binding to the untranslated regions (UTRs) of target mRNAs, resulting in RNA-induced silencing complexes (RISCs) that inhibit the translation of or degrade the target mRNAs. Some miRNA dysregulation is involved in the pathophysiological mechanisms of DKD. A transcriptomics study revealed that miR-15b-5p is responsible for apoptosis in mouse MCs, which promotes DKD progression and renal function deterioration. Furthermore, urinary levels of miR-15b-5p have a negative correlation with the estimated glomerular filtration rate (eGFR) and a positive correlation with the urinary albumin/creatinine ratio (uACR), indicating that it could be used as a predictor of DKD progression 96. Chen et al. 97 reported that miR-216a-5p was highly expressed in HG-treated MCs and promoted cell apoptosis. miR-21 is involved in renal fibrogenesis 98-100. Compared with healthy individuals, diabetic patients have greater concentrations of miR-21 in their blood and urine. A study revealed that miR-21 expression was considerably increased in STZ-induced DKD, which promoted podocyte motility and MC hypertrophy while also increasing the inflammatory response and fibrosis gene expression 101. miR-21 overexpression contributed to podocyte dedifferentiation and MC activation through the Wnt/β-catenin and TGF-β1/Smad3 pathways 102. Notably, the inhibition of several miRNAs also promotes fibrosis. For example, TGF-β1 inhibits miR-29 expression, leading to increased collagen expression and kidney fibrosis 103. These findings suggest that targeting miRNAs with pharmaceuticals could represent a unique and promising therapeutic approach for slowing the progression and consequences of DKD.

LncRNAs are noncoding RNAs that include more than 200 nucleotides. The existing evidence suggests that dysregulation of lncRNAs is linked to the development of a range of disorders, including DKD 104-106. On the one hand, lncRNAs can act through a variety of signaling mechanisms. For example, Chen et al. 107 discovered that the expression of the lncRNA SOX2OT was reduced in STZ-induced diabetic nephropathy mice and high-glucose-treated MCs, resulting in autophagy suppression, MC proliferation, and fibrosis-related gene expression via the Akt/mTOR pathway. Additionally, DANCR, a new lncRNA, inhibited TGF-β/Smad signaling in human renal MCs (HRMCs) 108. On the other hand, lncRNAs can act as miRNA sponges and indirectly affect protein expression. The lncRNA MIAT played a role in DKD progression by modulating the miR-147a/E2F3 axis 109. XIST, an lncRNA X inactivation-specific transcript, increases MC proliferation in DKD by inhibiting miR-423-5p and increasing HMGA2 expression 110. Silencing the lncRNA SNHG14 decreases MC proliferation and ECM accumulation by increasing the level of miR-424-5p and decreasing the level of SOX4 111. In contrast, Tu et al. 112 found that LINC01232 attenuated MC proliferation and fibrosis in DKD, which was achieved by attenuating MSH2 inhibition by miR-1250-3p.

**Figure 1:**
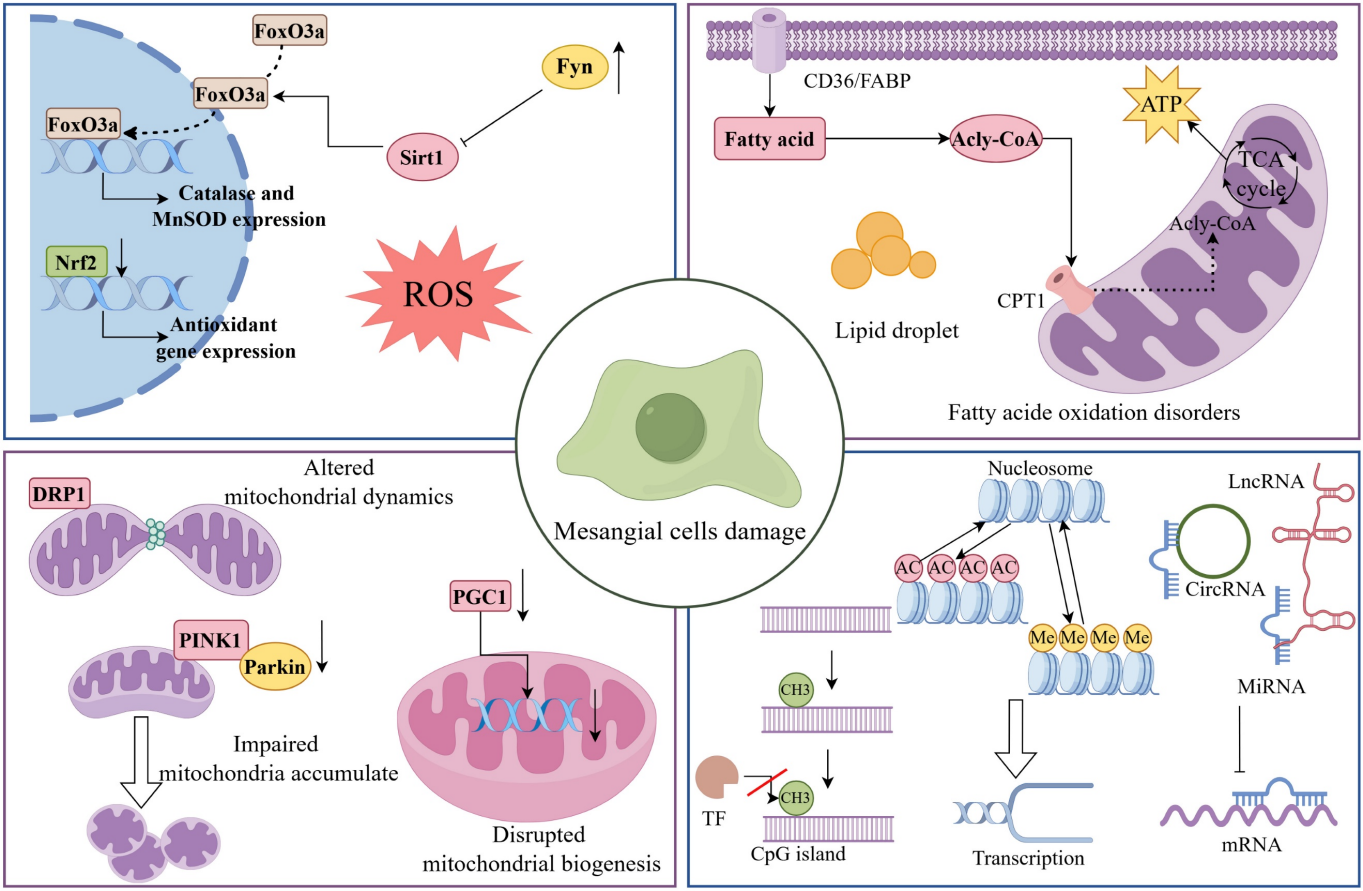
** Mechanisms of MCs damage in DKD.** During DKD, MCs undergo a range of pathological changes, including oxidative stress, mitochondrial dysfunction, abnormal lipid metabolism, and epigenetic dysregulation. As shown, Fyn expression was upregulated in DKD MCs, which inhibited the expression of antioxidant genes such as catalase through the Sirt1/FoxO3a pathway. In addition, Nrf2 was downregulated in HG-induced MCs, leading to ROS accumulation and inducing MCs injury. Dysfunctional mitochondria contribute to the progression of DKD. In DKD, impaired mitophagy, abnormal mitochondrial dynamics, and decreased mitochondrial production all contribute to MC destruction. It is worth noting that lipid metabolism undergoes substantial dysregulation in the early stages of DKD. Among other ways, poor fatty acid utilisation causes the production of lipid droplets, which has a negative impact on MCs. Finally, epigenetic alterations such as DNA methylation, histone modifications and non-coding RNA modifications are involved in MCs damage and subsequent dysfunction in DKD.

The circRNAs, which are noncoding RNAs with a circular structure, influence gene expression through the mechanism of competing endogenous RNAs (ceRNAs) and play significant biological roles in a variety of disorders, similar to lncRNAs. Several investigations have confirmed that circRNAs serve as molecular sponges for miRNAs in DKD 113, 114. For example, the circRNA DLGAP4 sponges miR-143 and regulates the ERBB3/NF-κB/MMP-2 axis, which in turn leads to the proliferation and fibrosis of MCs 115. Taken together, these findings indicate that noncoding RNAs play a variety of roles in DKD and are critical epigenetic regulatory molecules for early detection, targeted therapy, and prevention (Table 1) (Figure 1).

In addition to the characteristics discussed above, mechanical pulling is one of the mechanisms of early injury in MCs. Liu et al. reported that in DKD, glomeruli activate mechanosensitive transcriptional regulators, such as the transcription factor serum response factor (SRF) and coactivators myocardin-related transcription factor A and B (MRTFA/B) and yes-associated protein 1 (YAP1 or YAP) 116. MRTF-SRF was specifically activated in DKD MCs. Furthermore, a recent study revealed that YAP and TAZ were activated in MCs cultivated with high glucose, which significantly increased extracellular matrix deposition in hyperproliferating MCs 117. Activated YAP/TAZ bind to and increase the transcriptional activity of N-Myc proteins, which eventually leads to MC damage and DKD development. A groundbreaking study revealed a novel regulatory pathway underlying MC hyperproliferation in DKD. The research demonstrated that GABP is specifically overexpressed in DKD MCs, where it directly binds to the GLI1 gene promoter region to increase its transcription, thereby driving aberrant MC proliferation and ECM accumulation 118. This discovery provides fresh insights into the mechanisms of renal fibrosis.

## Crosstalk between MCs and other cells in DKD

In recent years, there has been an endless stream of research on the role of cellular interactions in the occurrence and development of DKD. MCs possess secretory functions and can secrete large amounts of growth factors and cytokines in response to stimuli such as hyperglycemia and hemodynamic changes. These secretions act on surrounding cells, including podocytes and endothelial cells, through a paracrine mechanism and can also diffuse into the renal tubulointerstitium through the bulbotubular junctions, causing tubulointerstitial damage. In addition, MCs themselves can also receive signals transmitted by cytokines secreted by other cells. The unique positioning and physiological functions of MCs warrant exploration of their role in cellular interactions.

### Crosstalk between MCs and podocytes

#### TGF-β1

TGF-β is upregulated in DKD and plays a role in matrix protein synthesis and fibrosis 119. TGF-β1 is a common isoform of TGF-β and a biomarker for type 2 DKD 120. Therefore, it is particularly important to study the role of TGF-β1 in DKD. Previous studies have reported that the interaction between MCs and podocytes plays an important role in IgA nephropathy 121. A previous study revealed that HG can cause MCs to secrete exosomes, which was confirmed by detection of the exosome markers CD63 and TSG101 122. Coincubation of these exosomes with podocytes resulted in reduced expression of the podocyte-specific proteins nephrin, podocin, and WT-1 122. The level of TGF-β1 in exosomes from MCs treated with HG significantly increased and induced podocyte apoptosis through the TGFβ1/PI3K-AKT signaling pathway 122. Inhibition of TGF-β1 in MCs significantly improved the damage to podocytes caused by exosomes from high-glucose-treated MCs 122.

### Crosstalk between MCs and endothelial cells

#### Platelet-derived growth factor B (PDGF-B)/PDGFRβ

MCs are in direct contact with endothelial cells. MCs have contractile properties and maintain the structure of capillaries through contraction. MCs carry PDGFRβ during development 123. During the development of human glomeruli, glomerular MCs are formed in the middle and late stages, and PDGF-B expressed by endothelial cells is key to the formation of MCs. It has been reported that mice lacking PDGF-B or PDGFRβ genes lack MCs in their glomeruli, indicating that the development and formation of glomerular MCs depend on PDGF-B and its receptor PDGFRβ 124, 125. Surprisingly, in mice lacking these genes, the glomerular capillary network collapsed and was replaced by a single dilated capillary loop 124, 125. Endothelial cells promote the development of MCs by secreting PDGF-B, which in turn supports and maintains the structure of the capillary network. Another study showed that the PDGF-B/PDGFRβ signaling pathway is activated in DKD, promoting the proliferation of MCs and aggravating the progression of DKD 17, 126. Inhibiting PDGF-B improves STZ-induced MC dysfunction in DKD 127.

#### Integrin αvβ8 and its ligand transforming growth factor β (TGF-β)

Integrin αvβ8 is abundantly expressed in the kidney and is located mainly in MCs. According to research reports, mice lacking integrin αvβ8 develop proteinuria, azotemia, and endothelial cell apoptosis 128. Latent TGF-β is the main ligand of integrin αvβ8. Integrin αvβ8 expressed by MCs binds and sequesters latent TGF-β, reduces the activation and release of TGF-β, and inhibits TGF-β-mediated damage in endothelial cells 128. These findings suggest that MCs maintain the stability of glomerular endothelial cells by regulating the release of TGF-β through integrin αvβ8. In addition, another study revealed that exosomes from endothelial cells treated with high glucose were enriched in TGF-β1 compared with exosomes from endothelial cells treated with normal glucose 129. Treating MCs with these exosomes activated TGFβ1/Smad3 signaling, mediating MC proliferation and increasing matrix protein production 130. These studies provide a basis for the interaction between MCs and endothelial cells to maintain glomerular structure and function.

#### Endothelin-1 (ET-1)

ET-1 is one of the most potent vasoconstrictor factors. Earlier studies revealed that coculture of HMCs and human umbilical vein endothelial cells (HUVECs) significantly reduced the expression of ET-1 and endothelin-converting enzyme (ECE-1) compared with culturing HUVECs alone 131. Studies have shown that endothelial cells treating with high glucose increases the secretion of ET-1, which activates the RhoA/ROCK signaling pathway by binding to the endothelin A receptor (ETAR) on MCs, thereby promoting MC proliferation and ECM accumulation 132-134. Endothelin B receptor (ETBR)-deficient DKD mice exhibit aggravated renal injury 132. Studies have shown that ET-1 combines with ETBR to inhibit the activity of NF-κB and reduce the secretion of ET-1 132.

#### Leucine-rich α2-glycoprotein 1 (LRG1)

LRG1 was formerly assumed to be involved in ocular neovascularization, but more recent research has revealed links to psoriasis, rectal cancer, atherosclerosis, and diabetic nephropathy 135-138. Previous research has indicated that LRG1 released by renal tubular epithelial cells enhances TGF-β-mediated Smad3 activation in renal fibroblasts, suggesting that LRG1 can mediate TGF-β signaling in a paracrine manner 139. In 2023, a cohort study by Liu et al. 137 reported that urinary LRG1 was related to a rapid deterioration in renal function and the development of massive albuminuria, providing evidence that LRG1 may be an essential component driving the progression of DKD. Furthermore, single-cell RNA sequencing (scRNA-seq) research by Tsuruta et al. identified LRG1 as a marker of kidney damage linked to fructose overconsumption in DKD 140. In DKD, LRG1 expression is increased in glomerular endothelial cells and is associated with glomerulosclerosis. The potential reason for this is that endothelial cells in a high-glucose environment synthesize and release secreted LRG1, which diffuses into the mesangial area and triggers TGF-β signaling 140, 141. LGR1 may play a role in overactivating TGF-β signaling in DKD glomeruli, making it an attractive target for preventing and treating TGF-β-related renal disorders.

#### SLIT2/ROBO1

Slit2, a secreted protein, was initially discovered to play a functional role in axon rejection in the central nervous system. Recent evidence suggests that it can promote angiogenesis by binding to the Robo1 receptor 142, 143. Notably, an intriguing study revealed that aberrant intraglomerular angiogenesis may be linked to Slit2/Robo1 signaling in the early stages of DKD. Slit2 was shown to be upregulated in HRMCs treated with HG. Additionally, Slit2/Robo1 signaling was triggered in HRGECs treated with HG-HRMC-CM. These findings indicate that Slit2 may facilitate crosstalk between HRMCs and HRGECs in a high-glucose environment and that the function of Slit2 is dependent on the receptor Robo1, as evidenced by the suppression of PI3K/Akt signaling activation in HG-HRMC-CM-treated HRGECs following Robo1 silencing 144.

#### VEGF-B/VEGFR1

A member of the VEGF family, vascular endothelial growth factor B (VEGF-B) has well-established metabolic regulatory roles 145. Current evidence has demonstrated that systemic inhibition of VEGF-B signaling significantly reduces glomerular lipid accumulation and ameliorates diabetic nephropathy phenotypes in murine models 146. However, the cell-specific expression patterns of VEGF-B in renal tissue, its precise molecular mechanisms in regulating renal lipid metabolism, and its impact on renal function in diabetic patients remain to be elucidated. Recent studies have elucidated a dual mechanistic role of VEGF-B in DKD: (1) MC-derived VEGF-B activates endothelial VEGFR1 through paracrine signaling, promoting fatty acid uptake and renal injury; (2) adipose tissue VEGF-B enhances lipolysis via hormone-sensitive lipase (HSL), increasing the number of circulating fatty acids that activate mesangial VEGF-B signaling, thereby establishing a pathological 'adipose-circulation-kidney' axis 147.

### Crosstalk between MCs and tubular epithelial cells

#### miR-92a-1-5p

Renal tubular injury is also a pathological characteristic of DKD. Proximal tubular epithelial cell (PTEC) injury occurs in the early stage of DKD 148. The interaction between glomeruli and tubules in DKD has been demonstrated multiple times 149, 150. However, the interaction between PTECs and MCs needs to be further studied. One study revealed that supernatants or exosomes derived from HK-2 cells treated with HG can cause endoplasmic reticulum stress and myofibroblast cell transdifferentiation (MFT) in MCs 151. Using RNA sequencing to analyze the miRNA profile of PTECs from normal individuals and type 2 DKD patients, we found that the level of miR-92a-1-5p was significantly increased in PTECs from DKD patients 151. Moreover, the mimic of miR-92a-1-5p significantly induced the MFT of MCs. A potential target of miR-92a-1-5p may be RCN3 because HG-cultured HK-2-derived exosomes reduce the protein expression of RCN3 in MC, and significantly promote the MFT and endoplasmic reticulum stress in MCs when RCN3 is deleted 151. The level of miR-92a-1-5p in the urine of DKD patients is positively correlated with the glomerular filtration rate and urinary protein level, which suggests that miR-92a-1-5p may be used as a biomarker of diabetic kidney damage and provide guidance for the clinical diagnosis and treatment of DKD patients.

### Crosstalk between MCs and macrophages

#### TGF-β1

**Figure 2:**
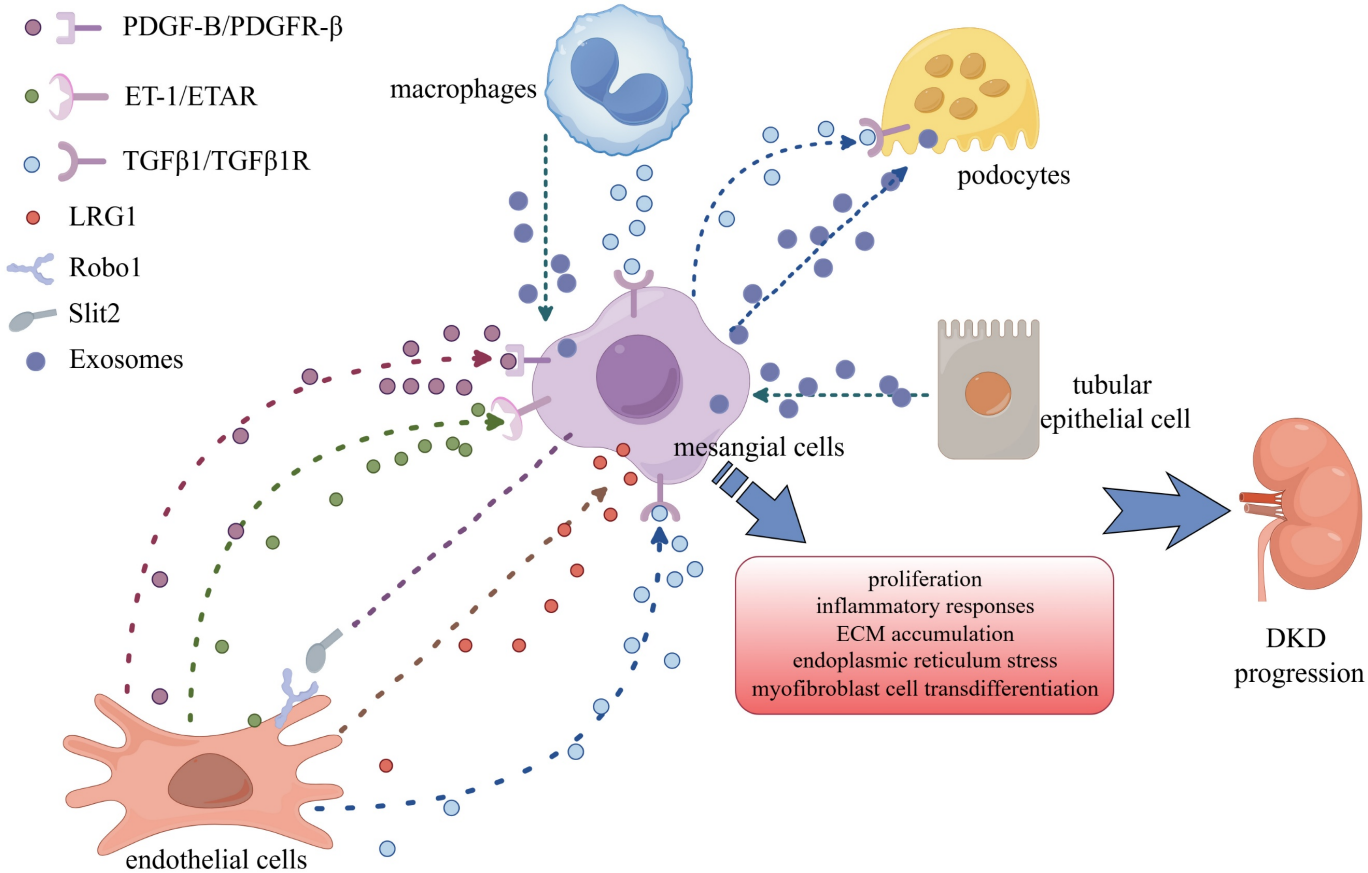
** Crosstalk between MCs and other cells in DKD.** MCs communicate with podocytes, endothelial cells, renal tubular epithelial cells, and macrophages via a variety of signalling chemicals to accelerate DKD progression.

Hyperglycemia activates resident renal macrophages; leads to the production of many chemokines and inflammatory factors; induces the recruitment of circulating monocytes/macrophages and renal cell damage; and promotes renal inflammation, fibrosis, and apoptosis 152. Coculture of macrophages and MCs under high-glucose conditions promotes the secretion of inflammatory factors and matrix proteins 152. When TGF-β-activated kinase-1 (TAK1) inhibitors are used, the secretion of inflammatory factors and extracellular matrix components is reduced by inhibiting the nuclear translocation of NF-κB p65 153. Macrophages treated with high glucose secrete large amounts of exosomes, which act on MCs and promote MC proliferation and activation. Mechanistically, high glucose induces macrophages to secrete TGF-β1, which activates MCs to produce extracellular matrix through the TGF-β1/Smad3 signaling pathway, promoting the progression of DKD 154 (Table 2) (Figure 2).

#### NLRP3 inflammasome

Activation of the NOD-like receptor 3 (NLRP3) inflammasome is involved in various kidney diseases through the regulation of proinflammatory cytokines such as IL-1β 155. For example, inhibition of the NLRP3 inflammasome can improve renal injury in DKD model mice 156. HG induces the activation of the NLRP3 inflammasome and NF-κB in HMCs, promotes the inflammatory response, and leads to the progression of DKD 157. Recent studies have not only shown that HG induces the M1 polarization of macrophages and promotes their secretion of exosomes but also that MCs internalize these exosomes 158. Exosomes from HG-treated macrophages promoted MC inflammatory responses and NLRP3 inflammasome activation and inhibited MC autophagy 158. Exosomes derived from HG-treated macrophages damage the kidney and induce mesangial hyperplasia *in vivo* 158.

## Therapeutic implications of MCs in DKD

### Pharmacological targeting of oxidative stress and inflammation in MCs

Previously, DKD treatment consisted primarily of lifestyle management, glycemic control, blood pressure control, proteinuria reduction, and the management of associated complications. Recent research has revealed several new pharmacological classes that have been shown to enhance renal outcomes in patients with type 2 diabetes, particularly sodium‒glucose cotransporter protein-2 inhibitors, mineralocorticoid receptor antagonists (MRAs), and selective endothelin receptor antagonists. Nonetheless, a considerable proportion of patients with diabetes inevitably show progression to DKD, and these therapeutic measures have provided modest support, encouraging exploration of the therapeutic potential of targeting MCs.

Oxidative stress and inflammation have emerged as critical factors in renal MC injury during DKD, coordinating their actions through multiple pathways, particularly the NF-κB and Nrf2 pathways. In a mouse model of DKD, activation of NF-κB promoted immune cell infiltration, the MC inflammatory response and fibrosis, and accelerated renal fibrosis 159. In OVE26 mice, Zhang et al. 160 found that pharmacological inhibition of PLK1 by the polo-like kinase 1 (PLK1) inhibitor BI-2536 lessened MC proliferation, alleviated proteinuria, and safeguarded renal function via NF-κB. Wang et al. 75 recently observed that ApoC3 can increase the phosphorylation of NF-κB and activate downstream inflammatory factors, including TNF-α, VCAM-1, and MCP-1, via TLR2 in MCs from T1DN mice, thereby exacerbating STZ-induced renal injury. Regrettably, ApoC3 deficiency did not seem to have a significant protective effect in STZ-induced DN mice, probably because there are already enough ligands for TLR2 in DN to exert an effect. In type 1 diabetic (T1DM) mice, dihydromyricetin (DHM), a flavonoid, inhibits NF-κB activation by binding to sphingosine kinase-1 (SphK1) and reducing its activity and protein expression, ultimately mitigating HG-induced expression of fibrosis and inflammatory molecules in GMCs 161. Zheng et al. identified another flavonoid, wogonin, that ameliorates renal inflammation and fibrosis by blocking the NF-κB and TGF-β1/Smad3 signaling pathways in MCs in DN 162. In addition, a cell-permeable peptide containing the kappa B kinase γ inhibitor /NF-κB essential regulatory factor-binding domain has been proven to ameliorate proteinuria and renal lesions 163. In addition, several compounds, such as icariin, eucommia lignans, β-caryophyllene and ampelopsin, can decrease HG-induced oxidative stress in MCs by activating the Nrf2/HO-1 pathway, hence slowing the course of experimental diabetic nephropathy and further emphasizing the importance of Nrf2 in the treatment of DKD 164-167. MCC950, an effective NLRP3 small molecule inhibitor, protects renal function by attenuating fibrosis in the HBZY-1 rat MCs line via inhibition of the NLPR3/caspase-1/IL-1β pathway 168. These findings support the notion that addressing the inflammatory response of MCs with medicines is an exciting avenue for the treatment of renal disease (Table 3).

### Pharmacological targeting of MC fibrosis

Considering that MC fibrosis and the secretion of large amounts of extracellular matrix components accelerate DKD renal damage, therapeutic strategies aimed at tackling the TGF-β pathway have aroused much attention. An isoquinoline alkaloid, epiberberine (EPI), was identified to ameliorate DKD by Xiao et al. Using protein-protein interaction (PPI) analysis and core gene screening, they found that EPI can dock with angiotensinogen (Agt) and disrupt the Agt-TGF-β/Smad2 pathway, thereby limiting MC proliferation and hypertrophy and ultimately ameliorating kidney damage in db/db mice 169. Recently, Li et al. 170 have also identified a Chinese herb, osthole, reduces HBZY-1 cell hypertrophy, ROS production, TGF-β1/Smad signaling pathway activation and ECM deposition, hence alleviating pathological renal damage in an STZ/high-fat/high-sucrose diet-induced rat model of type 2 diabetes mellitus. Inhibition of TGF-β signaling with EW-7197 attenuated fibrosis and inflammation in MCs, thus delaying the progression of DKD. Moreover, this intervention also diminished ROS production, ameliorated endoplasmic reticulum stress, and strengthened mouse kidneys against HG-induced damage 171. Barro et al. 172 synthesized a liposomal dosage form of puerarin to overcome its application limitations due to its water solubility. Liposomal puerarin reduced HG-induced TGF-β expression and Smad 2/3 nuclear translocation in RMCs. However, this impact has been confirmed only *in vitro*, and more research, including *in vivo* animal tests, is needed. Pyrrole-imidazole (PI) polyamides, a new biologic medicine, can block transcription by binding to the promoter regions of target genes. PI polyamides that target TGF-β1 have been developed to research chronic kidney disease, renal fibrosis, and DKD. In STZ-induced Wistar rats, PI polyamide treatment targeting TGF-β1 significantly alleviated proteinuria and pathological glomerular changes 173. In another study, HG promoted the nuclear translocation of transcription factor upstream stimulatory factor 1 (USF1), while USF1 PI polyamide significantly restricted the expression of TGF-β1 mRNA and protein, markedly reduced the expression of osteopontin, and hindered the conversion of MCs from the contractile to the synthetic phenotypes in the HG milieu, minimizing impaired renal function 174. Interestingly, viral mimetic nanoparticles (NPs) encapsulated with cinaciguat preferentially target MCs to regulate soluble guanylate cyclase (sGC) activity and 3′,5′-cyclic guanosine monophosphate (cGMP) generation. In injured kidneys, poor sGC function has been shown to promote MC proliferation and matrix accumulation 175, 176. Identifying compounds that improve mitochondrial dynamics as potential DKD treatments has been an emerging topic of interest. Several agents targeting mitochondrial function have been explored. For example, resveratrol inhibited oxidative stress and Drp1-mediated mitochondrial fission in diabetic renal MCs through the PDE4D/PKA signaling pathway, improving mitochondrial function 49. Furthermore, Lin et al. reported that tilapia skin peptides (TSP), a small-molecule mixture derived from tilapia skin, increases mitochondrial autophagic activity through the activation of Bnip3/Nix signaling, which lead to a reduction in mitochondrial ROS accumulation by scavenging damaged mitochondria and ultimately attenuates HG-induced fibrosis in GMCs 177.

Although the development of targeted anti-inflammatory and antifibrotic drugs remains in its early stages, clinical studies have demonstrated the protective effects of combined antioxidant therapy (N-acetylcysteine + taurine) in DKD. The trial data revealed that, compared with the placebo group, the treatment group presented an 18.6% reduction in the uACR, a 34.09% decrease in microalbuminuria, and a significantly greater improvement in the serum cystatin C level, suggesting that this regimen may delay DKD progression through multiple mechanisms 178.

### Certain clinical medications targeting MCs

Antihyperglycemic agents have been employed in the management of patients with T2DM and its complications, including DKD, for the past several decades. Metformin, the recommended glycemic control medicine, is widely utilized in clinical practice because of its ability to reduce hepatic gluconeogenesis and intestinal glucose absorption. At the mechanistic level, metformin delayed renal progression in STZ/high-fat diet-induced type 2 diabetic rats by decreasing oxidative stress in HG-exposed RMCs via the AMPK/SIRT1-FoxO1 pathway, increasing autophagy and alleviating hyperproliferation 179. Additionally, metformin partially reversed palmitate-mediated lipotoxicity-induced apoptosis in rat MCs. The underlying mechanism is likely that metformin stimulates GLP-1R expression, as agonists of GLP-1R alleviate the destruction of MCs under HG conditions; conversely, GLP-1R knockdown accelerates the apoptosis of MCs 180, 181. A recent randomized controlled trial demonstrated that semaglutide treatment significantly reduced the risk of the primary composite kidney outcome (including kidney failure [defined as the need for chronic dialysis or kidney transplantation or a sustained eGFR <15 mL/min/1.73 m^2^], ≥50% decline in the eGFR, or kidney/cardiovascular-related death) by 24% (HR 0.76, 95% CI 0.66-0.88; P=0.0003) in patients with type 2 diabetes and CKD, with 331 versus 410 first events occurring in the semaglutide and placebo groups, respectively 182. The abovementioned results not only emphasize the centrality of metformin in the treatment of DKD but also point out the utilization of GLP-1R agonists as an emerging therapeutic strategy in the prevention and treatment of DKD. Li and colleagues reported that GLP-1RA liraglutide treatment contributed to the shielding of MCs from hyperglycemia-mediated mitochondrial apoptosis, which had a beneficial influence on renal outcomes, supporting this assumption 183. Additionally, in cultured RMCs, liraglutide suppresses HG-stimulated production of fibronectin and collagen by augmenting Wnt/β-catenin signaling, which alleviates glomerular matrix accumulation and renal pathology abnormalities in DKD 184. Notably, several clinical trials have demonstrated that SGLT2 inhibitors may provide cardiovascular and renal benefits to patients with DKD in the form of a lower estimated GFR, a reduced risk of ESRD, and a lower rate of mortality due to cardiovascular effects 185-187. Toshinobu et al. recently showed that low dosages of canagliflozin reduced aberrant ROS production in HG-stimulated MCs and renal mesangial dilatation in db/db mice via the PKC/NADPH oxidase pathway, regardless of its glucose-lowering action, indicating that it has the unique ability to target MCs 188. Earlier studies have shown that SGLT2 inhibitors increase mitochondrial activity in diabetic vascular endothelial cells while decreasing oxidative stress injury, causing a reduction in vascular problems and an improved prognosis in type 2 diabetes patients 189. SGLT2 inhibitors may help protect against diabetic nephropathy-related cell damage by inhibiting mitochondrial dysfunction. In addition, the DPP-4 inhibitor selegiline has been shown to alleviate DKD by inhibiting TGF-β1/Smad signaling and upregulating heme oxygenase-1 (HO-1) expression in MCs 190, 191. Initial randomized controlled trials demonstrated that the DPP-4 inhibitor saxagliptin exerts potential renoprotective effects on DKD 192. However, the recent CARMELINA trial, which enrolled patients with both normal renal function and varying degrees of renal impairment, revealed that while linagliptin did not significantly improve the primary composite cardiovascular and renal endpoint (HR 1.04; 95% CI 0.89-1.22) compared with placebo, it significantly attenuated albuminuria progression 193. Prolyl hydroxylase structural domain (PHD) inhibitors have emerged as viable therapeutic agents for the treatment of chronic anemia in CKD because they activate HIF, which stimulates erythropoietin synthesis. When diabetic mice were given the PHD inhibitor enarodustat, proteinuria and glomerular damage were significantly reduced. The authors hypothesized that the renoprotective impact of enarodustat may be due to its ability to inhibit CCL2/MCP-1 production in MCs 194. Hypertension and proteinuria are also risk factors for promoting ESRD in people with type 2 DKD; hence, RAS inhibitors with dual effects of lowering blood pressure and hyperglycemia are a popular first-line treatments for DKD patients. RAS blockers include ACE inhibitors and angiotensin receptor blockers (ARBs). Telmisartan, an angiotensin II type 1 receptor blocker, has been shown to reduce AGE-induced MCP-1 in MCs and govern HG-induced TGF-β1 expression in HRMCs, underscoring the protective effect of RASi in MCs 195, 196. Furthermore, the SONAR trial, an international, multicenter, randomized controlled study involving 11,087 patients, demonstrated that the selective endothelin-A receptor antagonist atrasentan significantly reduced the risk of renal composite endpoints in patients with diabetes and CKD (HR 0.65; 95% CI 0.49-0.88) 197. Further research is warranted to determine whether the therapeutic value of the abovementioned medications for MCs may be extended to clinical application (Table 4).

## Conclusion and perspectives

MCs, a previously “forgotten” intrinsic glomerular cell, are receiving increasing attention due to their indispensable role in the course of DKD. Hyperglycemia in DKD causes damage to MCs via oxidative stress, mitochondrial failure, aberrant lipid metabolism, and epigenetic changes. These pathological changes accelerate the proliferation, hypertrophy, and phenotypic transformation of MCs in DKD, which in turn secrete a large number of cytokines, chemokines, and profibrotic factors, facilitating coordination and cooperation between MCs and other cells to promote DKD progression, ultimately leading to irreversible glomerulosclerosis and loss of renal function. Based on these pathological changes and damage phenotypes, it is clear that MCs will be the subject of extensive investigation in the future.

Although the mechanisms of harm to DKD MCs in DKD are classified into four types above, they are all intimately related and intersect with one another. In the pathological context of DKD, the hyperglycemic state and the progressive accumulation of advanced glycation end products (AGEs) jointly disrupt the redox homeostasis within MCs, leading to excessive generation of ROS and subsequently triggering oxidative stress. The overproduced ROS act as 'molecular scissors' that directly target mitochondria, resulting in an imbalance of mitochondrial dynamics and dysfunctional mitophagy. This severely disrupts the normal energy metabolism and quality control mechanisms of the cell. Moreover, MCs in patients with DKD commonly exhibit significant lipid metabolism dysregulation, characterized by markedly increased fatty acid uptake, excessive triglyceride accumulation, and disrupted cholesterol metabolic pathways. These aberrant lipid metabolites are far from 'inert bystanders'—instead, they drive alterations in epigenetic modifications through diverse molecular mechanisms. Notably, recent studies have revealed that sustained lipotoxic stimulation induces H3K9me3, an epigenetic marker that can be stably inherited during cell division, resulting in a 'metabolic memory' effect. Consequently, even after removal from the lipotoxic environment, cells retain dysregulated metabolic traits, persistently exacerbating DKD progression. These findings underscore the importance of comprehensively understanding the complex pathogenic mechanisms underlying MC injury in DKD. The ability of MCs to respond to innate immunity and stimuli by influencing immune cell recruitment via chemokine production highlights their role in numerous inflammatory responses and immunological-related disorders. Research has shown that MCs have significant phagocytic activity and can remove proteins that pass through the filtration membrane and collagen lost from the basement membrane, promoting basement membrane renewal and filtration barrier homeostasis. Currently, strategies for the prevention and treatment strategies of MCs damage in DKD involve two main pathways: anti-inflammatory and antifibrotic pathways. The most important signaling pathways are the NF-κB signaling and TGF-β pathways, which are activated by various stimuli in DKD, causing the proliferation and phenotypic transformation of MCs and the secretion of chemokines to mediate intercellular communication and the transmission of injury signals. In this review, various pharmacological molecules that act against MC inflammation and fibrosis are summarized, aiming to provide new perspectives for the clinical prevention and treatment of DKD. Furthermore, numerous commonly administered medicines, including the glucose-lowering drug metformin, GLP1R agonists, and SGLT2 inhibitors, have been demonstrated to improve mitochondrial performance in DKD MCs. Interestingly, the protective effect of SGLT2 inhibitors on thylakoid cells in DKD was not dependent on their hypoglycemic activity. This begs the question of whether there are other possible pathways for the positive effects of glucose-lowering medicines on MCs. Furthermore, drug safety concerns warrant equal attention. Although antioxidant therapy aims to mitigate oxidative stress by scavenging excessive ROS, overintervention in oxidative processes may disrupt the intrinsic redox homeostasis within cells, consequently leading to cellular dysfunction and even severe consequences such as increased tumorigenic risk. Therefore, in the development and application of therapies targeting MC injury in DKD, it is imperative to balance therapeutic efficacy with safety and comprehensively evaluate potential adverse effects.

At present, DKD is becoming increasingly common, and it is the leading cause of end-stage renal disease in developed countries. The complex pathogenesis of DKD is not favorable for its prevention and treatment. The main treatments for DKD include blood glucose lowering, proteinuria control, and renal function improvement. However, the incidence and mortality rates of diabetic nephropathy continue to increase. Therefore, in-depth study of the pathogenesis of diabetic nephropathy, the identification of new therapeutic targets, and the development of effective drugs are urgently needed for the current medical community to prevent and treat diabetic nephropathy. Future research is suggested to focus on two key directions: (1) elucidating novel mechanisms regulating MC proliferation, apoptosis, and ECM metabolism to uncover previously unidentified signaling pathways; and (2) utilizing single-cell multiomics technologies to decipher MC heterogeneity and identify critical pathogenic subpopulations, thereby providing new therapeutic targets for precision medicine approaches. In clinical research, optimizing existing therapeutic regimens and conducting novel drug trials are pivotal for improving outcomes in patients with DKD. Current pharmacological interventions targeting MCs, while demonstrating modest efficacy, remain limited in their therapeutic scope. Fundamentally, contemporary approaches to MC modulation persist at a 'broad-spectrum suppression' level—analogous to employing a fire extinguisher to quell an entire building when targeting a single flame. Future investigations must pioneer the development of 'molecular scalpels'—sophisticated modalities enabling single-cell precision targeting, spatiotemporally controlled drug delivery, and artificial intelligence-driven personalized therapeutic strategies to achieve precise regulation of pathogenic MCs.

## Figures and Tables

**Table 1:** DKD related non-coding RNA

non-coding RNA changes	Mediators or Related Pathways	Effects	Reference(s)
miR-15b-5p upregulation	Targeting BCL-2	Promoting DKD progression and deterioration of renal function	96
miR-216a-5p upregulation	Inhibiting MAPK pathway	Promoting MC apoptosis	97
MiR-21 upregulation	Activating the TGF-β1/Smad3 pathway	Promoting MC hypertrophy	101, 102
miR-29 downregulation	Targeting ECM components	Promoting collagen expression and kidney fibrosis	103
LncRNA SOX2OT downregulation	Activating Akt/mTOR pathway	Promoting MC proliferation, and fibrosis gene expression	107
LncRNA DANCR downregulation	Inhibiting TGF-β/Smad signaling	Inducing extracellular matrix accumulation in HMCs	108
lncRNA MIAT upregulation	Modulating the miR-147a/E2F3 axis	Inducing mesangial cell proliferation and fibrosis	109
lncRNA XIST upregulation	Inhibiting miR-423-5p	Increasing MC proliferation in DKD	110
lncRNA SNHG14 upregulation	Inhibiting miR-424-5p	Promoting mesangial cell proliferation and ECM accumulation	111
LINC01232 upregulation	Attenuating miR-1250-3p	Attenuating MCs proliferation and fibrosis in DKD	112
CircRNA DLGAP4 upregulation	Inhibiting miR-143	Leading to proliferation and fibrosis of MCs	115

**Table 2:** Crosstalk between MCs and other cells in DKD

Cell Types	Mediators or Related Pathways	Effects	Reference(s)
GECs → MCs	PDGF-B/PDGFR-β signaling pathways	Promoting the proliferation of MCs and aggravating the progression of DKD	127
GECs → MCs	TGFβ1/Smad3 signaling	Mediating MCs proliferation and increased matrix protein production	130
MCs→ GECs	Integrin αvβ8/TGF-β	Inhibiting TGF-β-mediated damage in endothelial cells	128
GECs → MCs	ET-1/ETAR	Promoting MCs proliferation and ECM accumulation	132
GECs → MCs	LRG1	Overactivating TGF-β and driving the progression of DKD.	141
MCs→ GECs	SLIT2/ROBO1	Promoting GECs aberrant intraglomerular angiogenesis	144
MCs→ podocytes	TGFβ1/PI3K-AKT signaling pathway	Induced podocytes apoptosis	122
MCs→ podocytes	Endoplasmic reticulum (ER)-associated degradation (ERAD)	Inhibiting of ERAD-related proteins, inducing podocyte apoptosis, and promoting the progression of DKD	198
PTECs → MCs	miR-92a-1-5p	Promoting endoplasmic reticulum stress and myofibroblast cell transdifferentiation (MFT) in MCs	151
Macrophages → MCs	TGF-β1/Smad3 signaling pathway	Promoting the proliferation of MCs and the progression of DKD	154
Macrophages → MCs	NLRP3 inflammasome	Activating of NLRP3 inflammasome and inhibiting MC autophagy	158

**Table 3:** Pharmacological moderators of MCs in DKD

Mediators or Agents	Mechanisms	Effects	Reference(s)
BI-2536	Inhibiting PLK1 and NF-κB	Lessening MCs proliferation, alleviating proteinuria, and safeguarding renal function	160
ApoC3	Increasing phosphorylation of NF-κB	Exacerbating STZ-induced renal injury	75
Dihydromyricetin	Inhibiting NF-κB activation by binding to sphingosine kinase-1 (SphK1)	Mitigating HG-induced expression of fibrosis and inflammatory molecules in GMCs	161
Wogonin	Blocking the TGF-β1/Smad3 signalling	Ameliorating renal inflammation and fibrosis	162
Cell-permeable peptide	Targeting NF-κB	Easing proteinuria and renal lesions	163
Icariin	Mediating p62-Dependent Keap1 Degradation and Nrf2 Activation	Decreasing extracellular matrix accumulation in MCs	166
Eucommia lignans	Mediating the AR/Nrf2/HO-1/AMPK axis	Alleviating the progression of diabetic nephropathy	165
β-Caryophyllene	Activating the Nrf2/HO-1 pathway	Decreasing HG-induced oxidative stress in MCs	167
Ampelopsin	Activating the Nrf2/HO-1 pathway	Inhibiting HG-induced extracellular matrix accumulation and oxidative stress in MCs	164
MCC950	Inhibiting of the NLPR3/caspase-1/IL-1β pathway	Protecting renal function by attenuating rat MCs line HBZY-1 fibrosis	168
Epiberberine	Disrupting the Agt-TGF-β/Smad2 pathway	Limiting MCs proliferation and hypertrophy, and ultimately ameliorating kidney damage in db/db mice	169
Osthole	Reducing TGF-β1/Smads signaling pathway activation	Inhibiting HBZY-1 hypertrophy, ROS production and ECM deposition	170
EW-7197	Inhibiting of TGF-β signaling	Attenuating fibrosis and inflammation in MCs	171
Liposomal puerarin	Reducing HG-induced TGF-β expression and Smad 2/3 nuclear translocation in RMCs	Protecting mesangial cells	172
Pyrrole-imidazole (PI) polyamides	Targeting TGF-β1	Alleviating proteinuria and glomerular pathological changes	173
USF1 PI polyamide	Restricting the expression of TGF-β1	Minimizing impaired renal function	174
Viral mimetic nanoparticles (NPs) encapsulated with cinaciguat	Regulating soluble guanylate cyclase (sGC) activity and 3′, 5′-cyclic guanosine monophosphate (cGMP) generation	Inhibiting MC proliferation and matrix accumulation	176
Resveratrol	Inhibiting PDE4D/PKA signaling pathway	Inhibiting oxidative stress and Drp1-mediated mitochondrial fission in diabetic renal MCs	199
Tilapia Skin Peptides	Activating the Bnip3/Nix signaling	Enhancing mitochondrial autophagic activity, scavenging damaged mitochondria in GMCs	177

**Table 4:** Clinical medications of MCs in DKD

Mediators or Agents	Mechanisms	Effects	Reference(s)
Metformin	Activating AMPK/SIRT1-FoxO1 pathway; stimulating GLP-1R expression	Enhancing autophagy and slowed down abnormal cell proliferation in high glucose cultured RMCs	179, 180
Liraglutide	Activating the ERK‑Yap signaling pathway and upregulating Sirt3 expression	Contributing to the shielding of MCs from hyperglycaemia-mediated mitochondrial apoptosis	183
Liraglutide	Enhancing the Wnt/β-catenin signaling	Suppressing production of extracellular matrix proteins and ameliorating renal injury of diabetic nephropathy	184
cagliflozin	Inhibiting the PKC/NADPH oxidase pathway	Reducing aberrant ROS production in HG-stimulated MCs and renal mesangial dilatation in db/db mice	188
Selegiline	Inhibiting TGF-β1/Smad signaling; upregulating heme oxygenase-1 (HO-1) expression	Alleviating DKD	190, 191
Enarodustat	Inhibiting the CCL2/MCP-1 production	Reducing proteinuria and glomerular damage	194
Telmisartan	Reducing AGE-induced MCP-1;governing HG-induced TGFβ1 expression	Alleviating DKD	195, 196

## Abbreviations

DKD: diabetic kidney disease
MC: mesangial cell
ESRD: end-stage renal disease
KF: kidney failure
ROS: oxygen species
GBM: glomerular basement membrane
Nrf2: nuclear factor erythroid 2-related factor
HIF-1α: hypoxia-inducible factor-1α
mROS: mitochondrial ROS
EPO: erythropoietin
Drp1: dynamite-related protein 1
ROCK1: Rho-associated coiled-coil kinase 1
PDE4: phosphodiesterase-4
PKA: protein kinase A
mtDNA: mitochondrial DNA
nDNA: nuclear DNA
AMPK: AMP-activated protein kinase
PGC-1α: peroxisome proliferator-activated receptor gamma coactivator-1α
NRF1: nuclear respiratory factor 1
GLP-1R: glucagon-like peptide 1 receptor
CD36: cluster of differentiation 36
FABP: FA-binding protein
CPT: carnitine palmitoyltransferase
FFAs: free fatty acids
TRPC6: transient receptor potential canonical channel 6
NFAT2: nuclear factor of activated T cell 2
LPA: lysophosphatidic acid
ChREBP: carbohydrate-responsive element-binding protein
ABCA1: adenosine triphosphate binding cassette transporter A1
ABCG1: adenosine triphosphate binding cassette transporter G1
SR-BI: scavenger receptor class B
LXR: liver X receptor
sHDL: synthetic high-density lipoprotein
ApoC3: apolipoprotein C3
Hmgb1: high-mobility group box 1
CHOP: C/EBP homologous protein
DNMTs: DNA methyltransferases
HMT: histone methyltransferases
EZH2: zeste homolog-2
OGT: O-linked N-acetylglucosaminyltransferase
HAT: histone acetyltransferase
FN: fibronectin
miRNAs: microRNAs
lncRNAs: long non-coding RNAs
circRNAs: circular RNAs
UTRs: untranslated regions
RISCs: RNA-induced silencing complexes
ACR: albumin/creatinine ratio
eGFR: estimated glomerular filtration rate
HRMCs: human renal MCs
ceRNAs: competing endogenous RNAs
MRTFA/B: myocardin-related transcription factor A and B
YAP1: yes-associated protein 1
SRF: serum response factor
PDGF-B: platelet-derived growth factor B
TGF-β: transforming growth factor β
ET-1: endothelin-1
HUVEC: human umbilical vein endothelial cells
ECE-1: endothelin-converting enzyme
ETAR: endothelin A receptor
ETBR: endothelin B receptor
LRG1: leucine-rich α2-glycoprotein 1
scRNAseq: single-cell RNA sequencing
VEGF-B: vascular endothelial growth factor B
PTEC: proximal tubular epithelial cell
MFT: myofibroblast cell transdifferentiation
TAK1: TGF-β activated kinase-1
NLRP3: NOD-like receptor 3
MRA: mineralocorticoid receptor antagonist
PLK1: polo-like kinase 1
T1DM: type 1 diabetic
DHM: dihydromyricetin
SphK1: sphingosine kinase-1
EPI: epiberberine
Agt: angiotensinogen
PI: pyrrole-imidazole
USF1: upstream stimulatory factor 1
NPs: nanoparticles
sGC: soluble guanylate cyclase
cGMP: guanosine monophosphate
TSP: tilapia Skin Peptides
HO-1: oxygenase-1
PHD: prolyl hydroxylase structural domain
ARBs: angiotensin receptor blockers
AGEs: advanced glycation end products
